# From Beer Pong to the OR: Management of Metallic Bottle Cap Foreign Body Ingestion

**DOI:** 10.7759/cureus.93955

**Published:** 2025-10-06

**Authors:** Richa S Nathan, Luke Mammen, Melissa Mortensen

**Affiliations:** 1 Otolaryngology - Head and Neck Surgery, Albany Medical College, Albany, USA; 2 Otolaryngology - Head and Neck Surgery, Albany Medical Center, Albany, USA

**Keywords:** airway management, esophageal inlet, foreign body ingestion, metallic bottle cap, rigid esophagoscopy

## Abstract

Foreign body (FB) ingestion is a common presentation seen by otolaryngologists ranging from mild discomfort to life-threatening airway compromise. Although there is a plethora of research in the literature describing the best approach to managing common FB ingestion, there is a paucity of literature describing the safe extraction of more rare FBs, such as metallic bottle caps. This case highlights the successful management of a metallic beer bottle cap lodged in the esophageal inlet and discusses key clinical considerations for atypical FB ingestions. A 19-year-old man presented with acute throat pain, odynophagia, and globus sensation after accidentally ingesting a bottle cap during a drinking game. Radiographs revealed a radiopaque disc at the proximal esophageal inlet. The FB was successfully removed via rigid esophagoscopy without complication. While many ingested FBs are benign and pass spontaneously, sharp, serrated, or irregularly disc-shaped objects such as metallic bottle caps pose a significant risk of mucosal injury, perforation, pressure necrosis, or obstruction. Young, inebriated men engaging in high-risk drinking behaviors are particularly vulnerable to accidental FB ingestion, warranting heightened clinical suspicion by ENTs. This case emphasizes the importance of prompt diagnosis, imaging, and airway evaluation in managing hazardous foreign bodies. Raising awareness about atypical FB ingestions and developing standardized protocols for their evaluation and treatment may improve patient outcomes and prevent life-threatening complications.

## Introduction

Foreign body (FB) ingestion is a common presentation seen by otolaryngologists, with presentations ranging from mild discomfort to life-threatening airway compromise. FBs account for 11% of the cases seen in otolaryngologic emergencies and carry potential for significant morbidity [1]. FB aspiration results in approximately 3,000 deaths in the US every year [2]. FB ingestion is most common in patients under the age of 15, with the highest incidence between one and three years of age [2].

Vegetative matter is the most common, but inorganic items such as coins and plastic toys are also commonly ingested [2]. Currently, there is a paucity of literature describing the safe extraction of rare FBs, such as metallic bottle caps. While many ingested FBs are benign and pass spontaneously, sharp, serrated, or irregularly disc-shaped objects such as metallic bottle caps pose a significant risk of mucosal injury, perforation, pressure necrosis, or obstruction, making them hazardous. Though infrequent, ingestion of such objects may be seldom recognized in certain populations [3]. Young adult men who engage in high-risk drinking behaviors, including binge drinking and social drinking games, may be especially vulnerable [3]. Alcohol intoxication impairs protective reflexes, increases impulsivity, and may contribute to accidental ingestion of dangerous objects during social activities [3]. Given this trend, it is important that otolaryngologists maintain a high index of suspicion when evaluating young, inebriated patients with FB ingestion. 

This case highlights the successful management of a metallic beer bottle cap lodged in the esophageal inlet and discusses key clinical considerations for atypical FB ingestions. The purpose is to share clinical pearls for identifying, imaging, and managing unusual FB ingestion. 

## Case presentation

**Figure 1:**
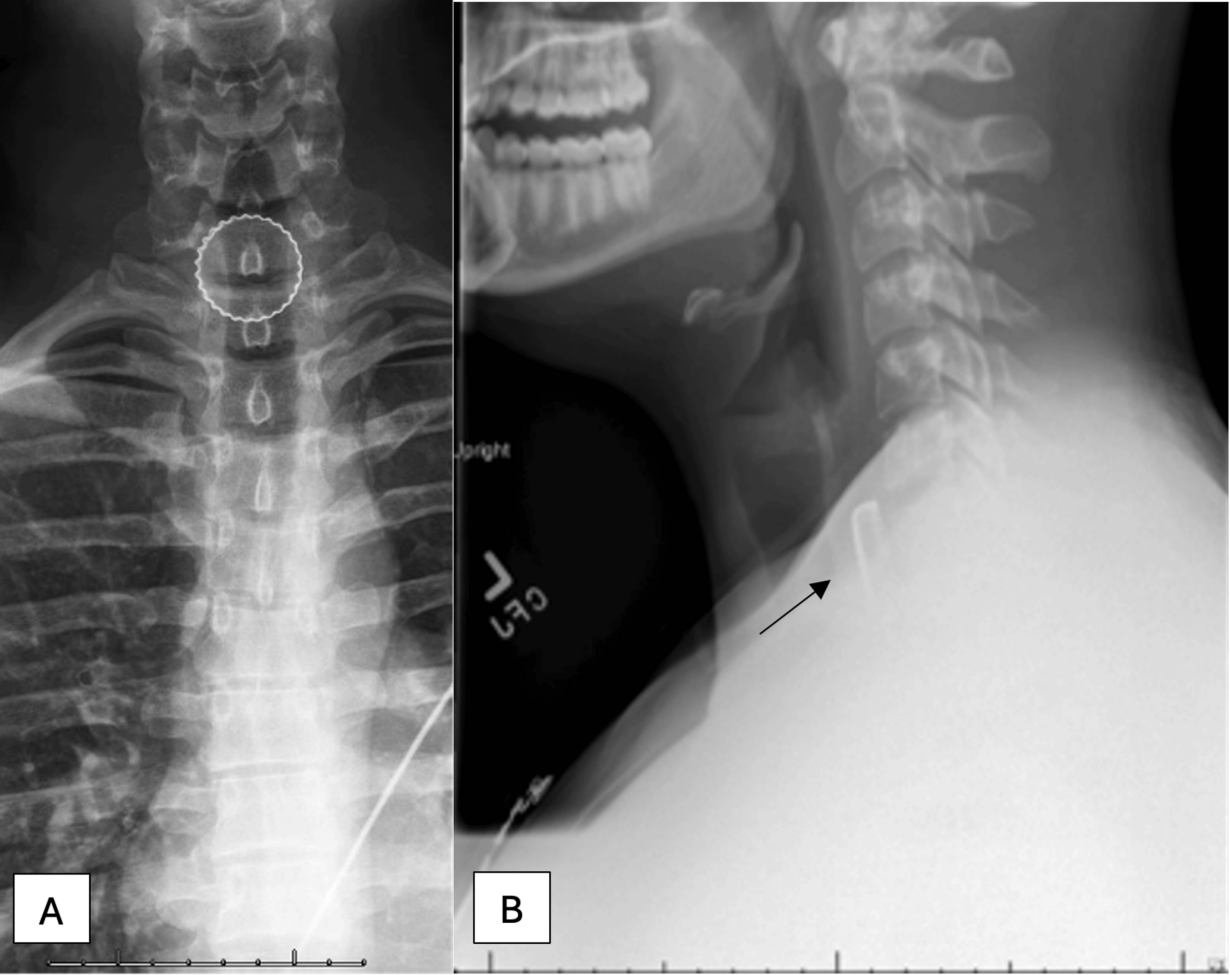
(A) Anterior-posterior and (B) lateral neck X-ray with a radiopaque foreign body at the esophageal inlet.

This case describes a 19-year-old man who presented to the emergency room with an acute onset of throat pain, odynophagia, dysphagia, and globus sensation secondary to the accidental ingestion of a metallic beer bottle cap during a party game. While playing a game of beer pong, the cap from a glass beer bottle had unknowingly fallen into his red solo cup prior to drinking from it approximately one hour prior to arrival. He denied shortness of breath, dysphonia, or hematemesis. Initial vital signs indicated mild hypertension (142/97 mmHg) and tachycardia (111 bpm), with a stable respiratory rate (18 breaths/min) and normal temperature (36.4 °C). The patient last ingested alcohol 30 minutes prior and was mildly inebriated. Physical examination revealed erythema of the posterior oropharynx without a visible FB, pooling of secretions, increased work of breathing, stertor, or stridor. A bedside flexible fiberoptic nasopharyngolaryngoscopy (FFNPL) revealed a patent airway with atraumatic laryngeal structures, no pooling of secretions, and no FB visible at the level of the oropharynx or larynx. A lateral and anteroposterior neck X-ray revealed a radiopaque, disc-shaped object consistent with a metallic bottle cap lodged at the proximal esophageal inlet, above the level of the clavicles (Figure 1).

**Figure 2:**
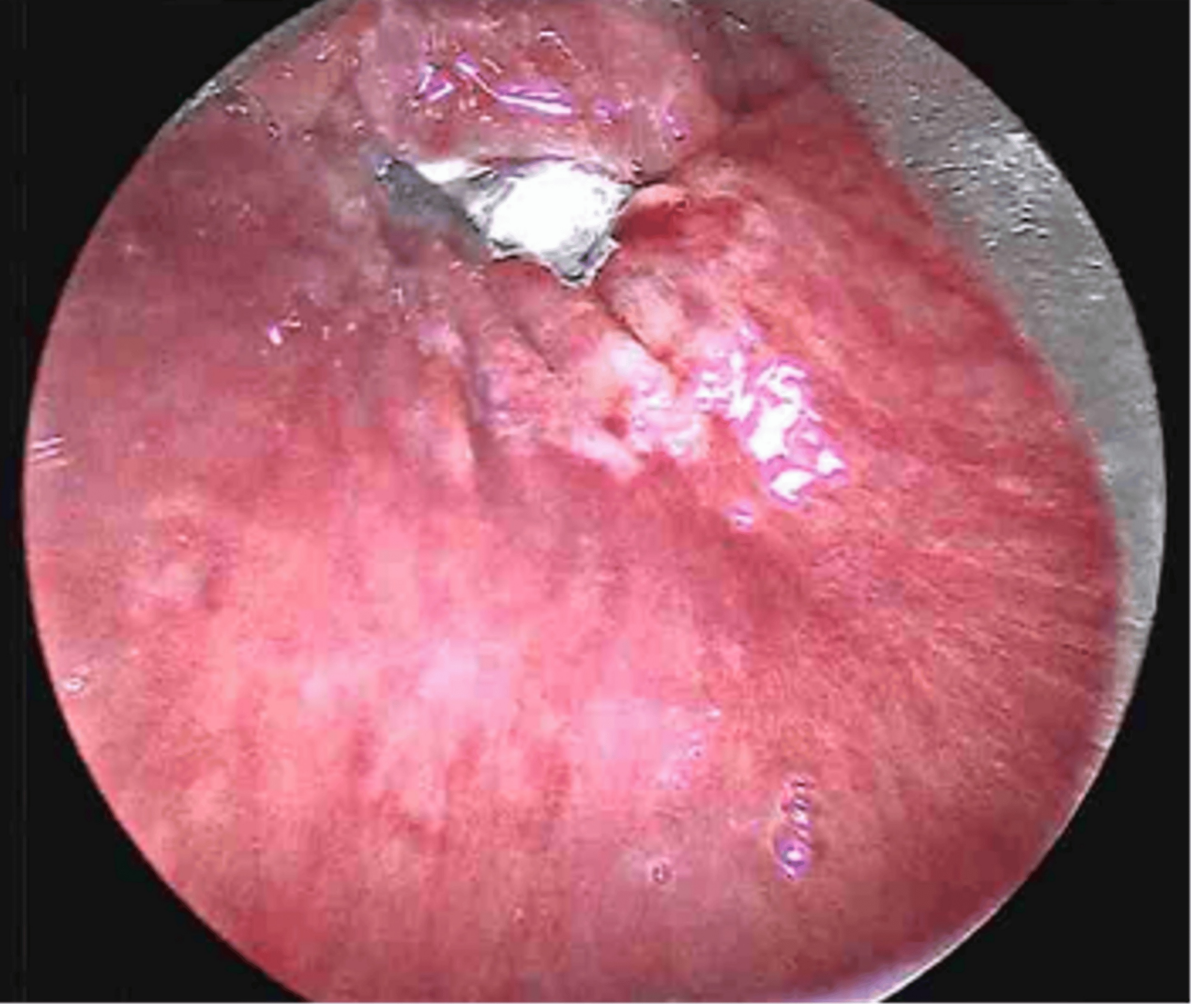
Esophagoscopy showing bottlecap at esophageal inlet with superficial mucosal tear posteriorly and early pressure necrosis anteriorly.

Despite having a stable airway, the patient required surgical intervention due to the FB's persistence at the esophageal inlet and potential for mucosal damage and even perforation. The patient was taken urgently for rigid esophagoscopy. After induction of general anesthesia and endotracheal intubation, a Dedo laryngoscope was inserted into the oral cavity and advanced to the esophageal inlet. Esophagoscopy revealed the bottle cap at the esophageal inlet with a small superficial mucosal tear posteriorly and early pressure necrosis anteriorly (Figure 2).

**Figure 3:**
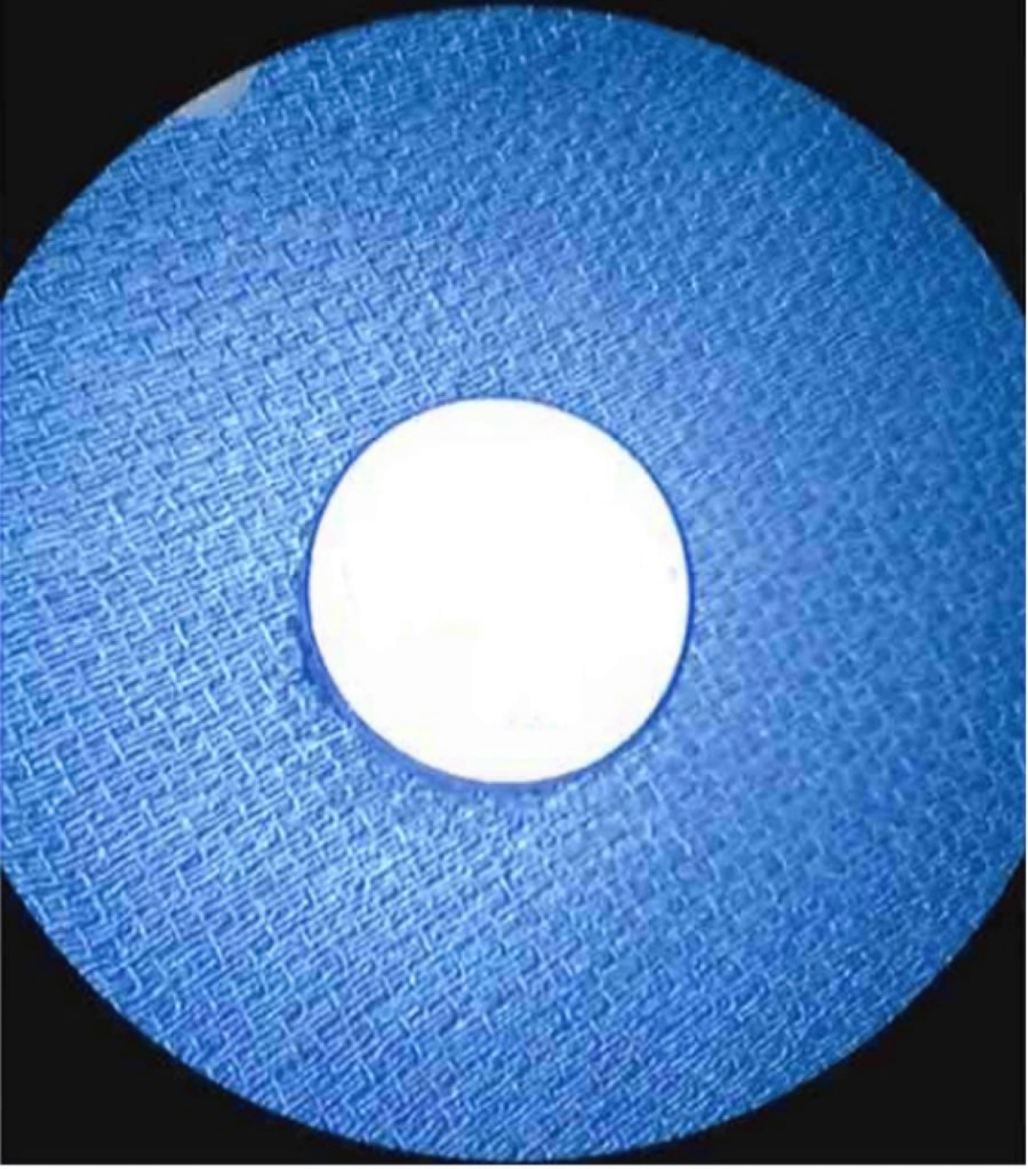
Metallic bottle cap foreign body.

The bottle cap was visualized and successfully removed using upward-angled cupped forceps without resistance. After removal, esophageal examination revealed the noted superficial mucosal tear and anterior pressure necrosis, without evidence of bleeding or perforation (Figure 3). 

The patient was extubated without complication and transferred to the post-anesthesia care unit in stable condition. He tolerated the procedure well and was discharged later that day on a soft diet with no immediate complications. Routine laboratory testing, including a complete blood count, revealed a normal white blood cell count and no evidence of systemic infection. Based on these findings, antibiotics were not prescribed. The patient followed up in the outpatient Otolaryngology clinic one week later with a normal physical exam and flexible laryngoscopy. There was no evidence of residual symptoms such as dysphagia or airway compromise.

## Discussion

FB ingestion in adults is relatively uncommon compared to pediatric populations, but when it does occur, it often involves food-related bolus impaction or intentional ingestion in psychiatric or incarcerated populations [4]. FB ingestion happens more commonly in adults with certain pathological changes of the gastrointestinal tract such as strictures (about 37%), malignancy (about 10%), esophageal rings (about 6%), and achalasia (about 2% of cases) [4]. The frequencies of swallowed FBs vary widely with the most commonly swallowed FBs in adults being fish bones (9-45%), bones (8-40%), and dentures (4-18%) [4].

In these cases of FB ingestion, FFNPL is the mainstay of initial evaluation and is often normal, although pooling in the pyriform fossae may be seen [2]. The initial management of esophageal FB obstruction, especially in the setting of food bolus impaction, is conservative. Antispasmodics such as Buscopan and hyoscine are also routinely employed [2]. Intravenous glucagon can be used as a lower esophageal dilator; however, the evidence for its efficacy is questionable [2]. Higher risk ingestion such as button batteries requires immediate removal due to the risk of severe injury to the esophageal mucosa from liquefactive necrosis, potentially resulting in perforation and mediastinitis [4]. If conservative management fails, an esophagoscope is passed under general anesthesia and advanced into the esophageal lumen until the FB is visualized [4]. If a distal FB is suspected, it can be removed or pushed through to the stomach using a flexible endoscope. 

With the rise of high-risk drinking behaviors, it is important for otolaryngologists to maintain vigilance when obtaining a history and evaluating young adults who may be especially vulnerable. Inebriated patients may have increased impulsivity and impaired protective reflexes, which puts them at higher risk for accidental FB ingestion [3]. Accidental ingestion of atypical foreign objects, particularly those with sharp, rigid, or serrated features such as metallic bottle caps, presents unique challenges for diagnosis and management. While most ingested FBs are radiolucent or pass spontaneously, sharp and metallic objects pose a significantly higher risk of mucosal injury, pressure necrosis, or perforation, particularly when lodged at high-risk anatomical narrowing such as the esophageal inlet. In this case, the patient presented with symptoms of globus sensation, pain, and mild respiratory difficulty, all nonspecific but common in esophageal impactions [5]. Radiographic imaging was instrumental in confirming the diagnosis, with the bottle cap clearly visible on plain X-ray as a radiopaque circular object. Importantly, flexible nasopharyngolaryngoscopy ruled out airway compromise and demonstrated no pooling of secretions, supporting a decision for early surgical intervention rather than urgent airway protection. 

While the case described here was managed successfully, it is important to recognize that metallic bottle cap ingestion is not an isolated phenomenon. A recent German case series similarly reported 14 cases of bottle cap ingestion over a 10-year period in a single university town, suggesting a seldom-recognized public health issue in high-risk populations [3]. Their findings revealed that while bottle caps are inherently sharp and serrated, the resulting injuries were generally limited to mild mucosal erosions and erythema, similar to this case. This relative lack of severe complications was attributed to early presentation and the population of young, otherwise healthy men [3]. The events were strongly associated with rapid consumption of large quantities of beer from open bottles in poorly lit environments, increasing the risk of accidental ingestion [3]. Based on this data, clinicians should counsel at-risk patients to avoid beverages with serrated bottle caps and to consider alternative packaging or drinking practices to prevent future occurrences.

## Conclusions

This case illustrates the successful removal of a metallic bottle cap from the esophageal inlet in a young male patient following accidental ingestion during recreational drinking. Metallic bottle caps pose significant risks including airway obstruction, mucosal injury, pressure necrosis, and esophageal perforation. Successful removal of the bottle cap underscores the importance of prompt airway assessment and imaging followed by surgical intervention. Raising awareness about atypical foreign body ingestions and developing standardized protocols for their evaluation and treatment may improve patient outcomes and prevent life-threatening complications. 
